# Paradigm Shift in Treatment Strategies for Second-Degree Burns Using a Caprolactone Dressing (Suprathel^®^)? A 15-Year Pediatric Burn Center Experience in 2084 Patients

**DOI:** 10.3390/ebj3010001

**Published:** 2021-12-23

**Authors:** Katharina Schriek, Hagen Ott, Mechthild Sinnig

**Affiliations:** 1 Pediatric Burn Center, Division of Pediatric Surgery/Pediatric Urology, Auf der Bult Children’s and Youth Hospital, 30173 Hannover, Germany; schriek@hka.de; 2 Division of Pediatric Dermatology, Auf der Bult Children’s and Youth Hospital, 30173 Hannover, Germany; ott@hka.de

**Keywords:** pediatric burns, conservative treatment, deep dermal scalding, caprolactone dressing, split thickness skin grafting, treatment costs

## Abstract

Background: Thermal injuries represent a highly relevant epidemiologic problem with 11 million individuals affected globally each year, of which around 2.75 million are children. Different approaches to the conservative treatment of second-degree burns have been widely discussed in the existing literature. One method that has attracted increasing attention is the use of caprolactone dressings. This paper describes a study involving the therapeutic management of 2084 pediatric patients suffering from mixed superficial and deep dermal second-degree burns who received comprehensive expert treatment using caprolactone membranes at the pediatric hospital AUF DER BULT. Methods: A retrospective study was conducted to evaluate the frequency and effect of caprolactone membrane usage on children who were admitted to the pediatric hospital between 2002 and 2016 with mixed second-degree burns. The number of dressing changes under general anesthesia and the requirement for split thickness skin grafting were monitored and recorded. In addition, a cost comparison analysis of different treatment modalities was performed. Results: This retrospective study involved 2084 children who had been treated for mixed superficial and deep dermal burns between 2002 and 2016 using either caprolactone dressing (Suprathel^®^) (study group; n = 1154) or an alternative dressing material (control group; n = 930). Of the patients in the study group, 91.74% (n = 1053) were treated conservatively compared to 76.05% of the control group patients, meaning that 8.26% (n = 101) of the study group patients required skin grafting, compared to 23.95% (n = 223) in the control group. Additionally, the number of procedures under general anesthesia per patient was found to be 54.3% lower among all patients treated with caprolactone dressing (1.75 procedures per patient) compared to the entire control group (3.22 procedures per patient). In the subgroups, patients treated conservatively with caprolactone dressing required 1.42 procedures per patient compared to 2.25 procedures per patient in patients with alternative wound treatment. When split thickness skin grafting was necessary, 1.2 times as many procedures were performed on patients with alternative dressing compared to those treated with caprolactone dressing. Finally, the cost per patient was considerably lower in the conservative therapy group in comparison to the group that consisted of patients undergoing operative therapy with split thickness skin grafting. Conclusions: Caprolactone dressings were found to be beneficial for children who reported with mixed superficial and deep dermal burns. Specifically, they reduced the need for skin transplantation, the number of dressing changes under general anesthesia, and the treatment costs.

## 1. Introduction

Globally, thermal injuries are sustained by approximately 11 million individuals each year, 25% of whom are under 16 years old [1]. Hence, thermal injury should be regarded as a serious epidemiological problem [2]. Scalding injuries to toddlers are frequently recorded among household accident statistics, particularly as a result of cups containing hot drinks being knocked over or, in less developed countries, due to the practice of cooking over open fires [2,3]. In addition to flame burns, scalding can be categorized as a severe injury due to the high heat transferring capacity [4]. The care of burns victims is complex, consisting not only of acute therapy at the time of injury but also requiring years of conservative follow-ups. Both the complexity and duration of care tend to be frequently higher in pediatric cases, as numerous corrective interventions may be required due to the range of secondary problems that can occur in growing children, e.g., functional constraints caused by contracting scars. Moreover, the cost-effective management of pediatric burns injuries continues to be a challenging factor for burns units. Numerous studies have been undertaken to determine the relative merits of various conservative therapeutic approaches to second-degree burns. Under standard treatment that adheres to both national and international norms, the devitalized tissue is cleaned and debrided. Then the focus shifts to preventing infection, ensuring the best conditions for a scar-free wound healing, and reducing split-thickness skin grafting. In general, wound healing periods with less than 21 days are aimed for to achieve scar-free healing [5]. The caprolactone dressing Suprathel^®^ is a synthetic copolymer that consists of polylactide acid, trimethylenecarbonate, and e-caprolactone. Its high moisture permeability prevents the accumulation of wound fluid, thereby supporting wound healing and reepithelization. By reducing the pH level, it inhibits proteases and bacterial growth. Additionally, it stimulates the wound healing process by activating angiogenesis, fibroblast migration, and collagen synthesis. The dressing’s transparency after application to the wound allows good wound inspection. Due to its biodegradability, the caprolactone dressing does not need to be removed before the full reepithelization of the burn wound [6,7]. In all these procedures, effective pain relief should always be a core element, particularly for children. Accordingly, general anesthesia is frequently applied where pediatric burns patients are being treated [8]. The aim of the present study was to explore the outcome of this therapeutic approach in partial-thickness pediatric burns. An assessment was made using objective criteria, such as the number of dressing changes under general anesthesia and the incidence of skin graft procedures.

## 2. Materials and Methods

### 2.1. Patients

Patient files of infants, toddlers, and children younger than 17 years of age with second-degree burns, treated at our Pediatric Hospital between 1 January 2002 and 31 December 2016, were investigated in this retrospective study. In accordance with the above-mentioned criteria, only patients with second-degree (i.e., superficial and deep dermal) burns were included. The presence of additional third-degree burns or other severe trauma meant exclusion from the study.

Wounds were assessed by experienced pediatric burns surgeons, with severity defined according to TBSA (total body surface area) and depth (first degree, second degree [superficial and deep dermal], and third degree [9]). Each patient was classified and supervised by a burn expert.

### 2.2. Control Group

The standards of second-degree burns care in the control group required the debriding of devitalized tissue under general anesthesia, then applying materials such as silver-containing dressings, hydrocolloids, biosynthetic wound dressings, absorbent foam dressings, hydrofiber dressings, non-adherent lipidocolloid dressings, fatty gauzes, or dexpanthenol-containing cream. Depending on the wound healing process, the wound’s condition, and the ointment and dressing material, dressing changes were performed with or without general anesthesia. Decisions on whether to continue with conservative therapy or move on to split-thickness grafting were made between days 10–21.

### 2.3. Study Group

As with the control group, a similar debridement procedure under general anesthesia was carried out. Once completed, the caprolactone membrane (Suprathel^®^; PolyMedics GmbH, Denkendorf, Germany) was applied, adhering to the wound bed. To prevent adherence to the overlaying dressing, this was covered with a fatty gauze. Regular dressing changes of the overlaying dressings were performed every second to fifth day with or without anesthesia. According to the reepithelization process, decisions on skin grafting were made between days 10–21.

### 2.4. Cost Analysis

Using the 2016 data, an average comparative cost analysis was performed for each patient to compare two therapies: a therapy with only caprolactone dressing (n = 10) and a procedure with split thickness skin grafting (n = 10). The groups were matched according to age and gender, burn depth (2b°), and TBSA. Adjustments were made to exclude patients with relevant secondary diagnoses (e.g., respiratory infection, pneumonia, wound infection, disabilities), additional procedures (e.g., blood transfusion), intensive care needs, or difficult social circumstances that led to extended stays. The total care costs were calculated by our hospital’s medical control department, based on the German InEK (Institute for the Hospital Remuneration System) calculation scheme. The allocation keys specified by InEK were then utilized to apply for overhead costs. The InEK cost matrix provides an overview of a German Diagnoses Related Groups’ (DRG) case-related costs. Divided into cost types (personnel costs, material costs, infrastructure costs) and cost centers (including normal and intensive care wards, operating theater area, the anesthesia department, laboratory diagnostic areas, therapeutic procedures, and patient admission), it enables the costs incurred for a patient’s case to be allocated precisely. The costs were then compared with the DRG revenues generated. The DRG depends on diagnoses, procedures, age, sex, discharge status, and the presence of complications or comorbidities. The resulting costs and revenues were then compared according to the conservative and operative groups.

### 2.5. Statistical Analysis

SPSS database version 25 (SPSS Inc., Chicago, IL, USA) was utilized for the current research. Both t-test and chi-square test were used to compare categorical data between the study and the control groups, with two-sided *p* values < 0.05 considered as statistically significant. Correlation analyses and an ANOVA test were used to assess the association of number of procedures under general anesthesia with various parameters. There was a *p* value of <0.05 as a level of significance and a 95% confidence interval.

## 3. Results

In total, 2084 cases met with the inclusion criteria. In the predominately male group (57% male), patients ranged in age from 0 months to 17 years, with 80% of the total sample being under 5 years of age. The Pearson chi-square test was run and no significant difference was found in terms of age, sex, treatment, or affected TBSA (*p* < 0.05) between the study and control group. Ninety percent of all patients had a TBSA of <10% (Table 1). Figure S1 provides detailed results regarding the annual contribution of patients to the study and control groups.

### 3.1. Wound Treatment

**Figure 1:**
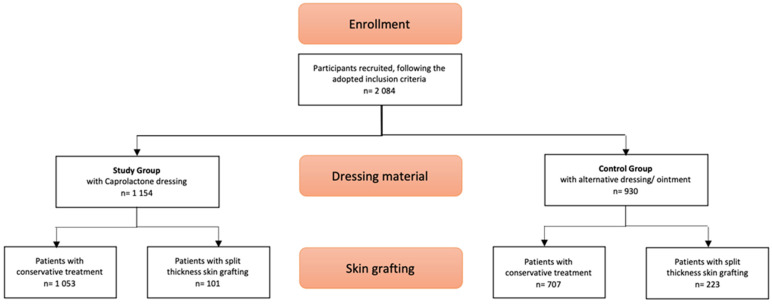
Flow diagram of participant selection, dressing materials, and necessity of skin grafting.

There were 1154 patients (55.5% of the total sample) included in the study group (Figure 1). Figure S2 presents a list of dressing materials used in the non-caprolactone control group. Of the study group, 91.74% (n = 1053) was treated conservatively, compared to 76.05% (n = 707) of the control group (see Figure 1). The type of dressing (caprolactone versus an alternative material) was found to be statistically significantly associated with the need for split-thickness skin grafting (*p* = <0.0001).

### 3.2. Procedures under General Anesthesia per Patient

The mean number of procedures under anesthesia per patient was found to be 54.35% lower among all patients treated with caprolactone dressing than the entire control group, with a *p*-value of <0.0001 (Table 2). With regard to conservative treatment, the mean number of procedures per patient differed significantly between the study and control groups, at 1.42 (min: 1; max: 6; SD: 0.84) and 2.25 (min: 0; max: 8; SD: 2) procedures per patient, respectively (*p* < 0.0001). The same was true for the number of procedures per patient under general anesthesia for split-thickness skin grafting, namely, 5.36 (min: 1; max: 9; SD: 1.87) and 6.40 (min: 1; max: 12; SD: 2.44) interventions per patient in the study and control groups, respectively (*p* < 0.0001).

A regression analysis was used to assess the association of procedures per patient with various parameters (adjusted r square 0.397). Biosynthetic wound dressing (Biobrane^®^) [OR (95% CI) = 1.563 (0.83–2.296)], silver sulfadiazine (Flammazine^®^) [OR (95% CI) = 2.349 (2.073–2.625)], hydrocolloids [OR (95% CI) =1.103 (0.879–1.326), hydrofiber dressing (Aquacel^®^) [OR (95% CI) = 1.043 (0.149–1.937)], and absorbent foam dressing Mepilex^®^ [OR (95% CI) = 2.041 (1.576–2.506)] were found to be the significant factors for increased number of procedures per patients under anesthesia. A significantly decreased number of procedures under anesthesia was seen in caprolactone dressing (Suprathel^®^) [OR (95% CI) = −2.985 (−5.698–0.273)]. The gender and age were not statistically significantly associated with the number of procedures under anesthesia.

### 3.3. Cost Analysis between Conservative and Operative Treatment

Costs per patient were considerably lower in the conservative therapy group when compared to the operative therapy with split-thickness skin grafting (Table 3). The difference between costs and revenues resulted in an average plus of €433.80 per patient for the conservative therapy procedure and an average loss of €−536.19 per patient for the operative therapy procedure.

## 4. Discussion

Since the 1970s, the use of silver sulfadiazine has been the gold standard for topical treatment of superficial and small, deep dermal burns. However, its silver-containing antimicrobial activity has induced rapid cell death and requires dressing changes at short intervals. As a result, a wide range of synthetic, biological, and biosynthetic skin substitutes was developed. Synthetic skin substitutes such as polyurethane and hydrocolloids are only useful for small and superficial second-degree burns [5,10,11]. In contrast, superficial and deep dermal wounds are the main reasons for biological dressings, but they carry the risk of viral contamination and allergic reactions. Moreover, biosynthetic dressings are known to enhance the natural wound healing process. In contrast with former products, caprolactone dressings positively influence wound healing by stimulating fibroblast migration and angiogenesis, supporting collagen synthesis [12], and reducing pH levels [13].

One main finding of our current investigation was the clear correlation between caprolactone dressing usage and the necessity for procedures to be carried out under general anesthesia. Specifically in the conservative treatment, dressing changes per patient under anesthesia were 1.58 times more necessary when no caprolactone dressing was used. Considering that the first intervention for the patients treated with caprolactone dressings had always been performed under general anesthesia due to the necessity of intensive wound debridement, most patients only required this single procedure under general anesthesia when treated conservatively with the caprolactone substitute. This number was lower in comparison to Cattelaens’ study, employing a nanocellulose-based wound dressing for mixed superficial and partial thickness pediatric burn wounds, in which the frequency of dressing changes under anesthesia was 2.4 times more on average per patient [14]. Likewise, Koehler’s retrospective study on negative wound pressure dressings in partial thickness burn injuries in children showed on average 3.5 times more dressing changes with intravenous narcotics or general anesthesia [15].

Our retrospective study was unable to systemically analyze recorded pain or infection rates due to a lack of data. Nonetheless, the literature showed that caprolactone dressings are more beneficial in terms of pain reduction compared to alternative materials such as absorbent foam dressings (Mepilex Ag^®^) and hydrophilized polyurethane (Omiderm^®^) [6,16,17,18]. In addition, Blome-Eberwein found an average pain level of 1.9 on a 10-point scale in superficial and deep second-degree burn wounds in adults and children; the author attributed the low score to the flexibility of the caprolactone-based substitute and its favorable wound milieu, with a pH close to physiologic skin pH [19]. Additionally, this might also contribute to the prevention of bacterial growth, which may explain the low infection rate of under 4% for mixed second-degree scald injuries presented in the literature [20]. As the caprolactone membrane molds to the wound bed, this removes the need for manipulation of the wound itself during dressing change procedures. Furthermore, only the outer compress dressing needs changing, facilitating easier dressing changes on the ward or in outpatient settings. Additionally, the membrane becomes transparent, allowing for an adequate wound assessment before it detaches during reepithelization [6,17,21]. Accordingly, other researchers, such as Schwarze and co-workers, agreed that the combination of pain reduction and a lower number of dressing changes present the major advantages of caprolactone dressing [6]. Considering the benefits offered by caprolactone dressings and the requirements for anesthetic procedures to implement wound debridement, the question of whether all thermal injuries are suitable for this treatment is of great importance. Our current treatment protocol recommends the application of caprolactone dressing once burns have reached 1.5% TBSA, with exceptions being burns located on the face, hands, and feet. In our opinion, injuries in these functionally and psychosocially crucial locations should also be considered for treatment with caprolactone dressing, even with less than 1.5% TBSA burns.

In our large pediatric burn patient population, the highly significant correlation between the use of caprolactone dressing and the reduced necessity for split thickness skin grafting is another key finding: Only 8.26% of the patients treated conservatively with the caprolactone dressing required grafting, compared to 23.95% of those treated with alternative dressings.

Our results are similar to those of Rashan et al. [21], who assessed the usability and effectiveness of the same caprolactone dressing in pediatric burns of partial thickness. In this comparatively small cohort of pediatric patients, 14% required skin grafting due to no expectation of spontaneous wound healing. In other studies, within which pediatric burns were treated with a variety of synthetic dressings, the number of required skin grafts was considerably higher (5 out of 21 (17%) and 3/17 (24%)) [22]. This also included the recently available nanocellulose-based wound dressing (Epicite hydro^®^), which shows a grafting rate of 12% [14] as well as the biosynthetic temporary skin substitute (Biobrane^®^) in mixed partial thickness burns in children. Hubik emphasized that 29% of the patients treated with a biosynthetic temporary skin substitute (Biobrane^®^) had to undergo further surgery after failure of the initial treatment [23]. Our research had similar outcomes to those reported by Keck et al. (n = 18 patients), who proposed that caprolactone dressings should be considered a viable alternative to split-thickness skin grafts when treating deep dermal burns [15]. Furthermore, Uhlig (n = 22 patients) also found that caprolactone dressings can lower the necessity for grafting [16,17].

Many investigations calculated the cost by using the sole price of the dressing material [24], which did not sufficiently reflect the complexity of the cost structure (theater staff, ward costs, etc.). Additionally, due to the differences in healthcare systems, it was difficult to compare our findings with other international studies that did include a broader range of cost structures. However, a British survey estimated the typical health compared care costs of a ≥2-day admission for a ‘pediatric burn’ at £2000–£3000 [25]. This corresponded with our own observations, according to which the conservative management of a mixed 2b° thermal injury costs an average of €3000. Nevertheless, our data suggest that conservative treatment with caprolactone dressing may lead to significant overall inpatient hospital care cost savings when compared to surgical therapy with split thickness skin grafting.

Our research’s methodological strengths include the large size of the investigated patient cohort (n = 2084), the standardized therapy algorithm in this single-center study, and the fact that there were no significant variations of demographic or disease characteristics between the two compared groups. The current study also has its limitations, however. This retrospective, descriptive study was based on the long-term observation of two groups characterized by a treatment regimen (caprolactone vs. alternative dressing materials), rather than an active comparison of two prospective intervention groups. This raises the issue of a potential treatment selection bias. For example, the burn team’s increasing experience with the properties of the caprolactone dressing over the investigated period may have also had a certain influence on the decision regarding split thickness skin grafting and the number of procedures under anesthesia, although this investigation did feature a high degree of consistency over a long period such as an institutional burn treatment protocols and a supervising burns expert team. Secondly, this research did not establish fixed protocols for assigning patients to either the caprolactone dressing or non-caprolactone dressing groups, though it was confirmed that the outcomes did not reveal any significant variations in gender, TBSA, or age between the different groups. Thirdly, additional outcome parameters could have been taken into consideration, including time spent in hospital and the cosmetic and/or functional outcomes for patients. However, it was shown, with regards to time spent in hospital as an inpatient, that there were many different factors that influenced this variable, including patients’ social circumstances, willingness to comply with treatment, and/or how far the patient lived from the hospital, among others. Because of insufficient documentation, functional and/or aesthetic outcomes could not be objectively compared in retrospect. Finally, regarding the cost analysis, only a small cohort of comparable patients with conservative versus operative therapy from a single year was examined. It was not possible to compare two conservative therapy groups with regard to the costs due to the retrospective, descriptive nature of the study as both the material costs and the DRG system revenues are subject to constant modification, while the hospital financing modalities change annually.

## 5. Conclusions

To summarize, this research represents a unique study covering a 15-year timeframe, within which 2084 pediatric patients suffering from mixed superficial and deep dermal second-degree burns received comprehensive expert treatment in one burns center. Caprolactone dressings were shown to be beneficial for mixed superficial and deep dermal burns in children. The necessity of skin grafting was reduced by 15.69% compared to alternative dressing materials. The number of procedures needed to be carried out under general anesthesia was found to be 54.35% lower among patients treated with caprolactone dressing compared to those treated with alternative dressing materials.

## Figures and Tables

**Table 1:** Gender, age groups, and affected TBSA in children with caprolactone dressing (study group) versus children without caprolactone dressing (control group).

Variables	Study Group n = 1154	Control Group n = 930	*p*-Value
male: female ratio	653/501 (1.4:1)	536/394 (1.3:1)	0.98
<5 years	964	711	0.61
>5 years	190	219	
TBSA			
<10%	1004	884	0.72
10–20%	117	31	
20–30%	20	12	
30–40%	8	5	
40–50%	1	2	

**Table 2:** Number of procedures under anesthesia.

Wound Treatment	Study Group n = 1154	Control Group n = 930	Significance
Conservative Treatment	1495 (29.59%)	1590 (28.25%)	*p* < 0.0001
Skin Grafting	541 (10.60 %)	1427 (31.48%)	*p* < 0.0001

**Table 3:** Annual average cost comparison per patient: conservative treatment in comparison to split thickness skin grafting.

	Conservative Therapy n = 10	Operative Therapy (with Skin Grafting) n = 10
average expenses per patient in €		
Mean	3755.56	14,383.98
Min	2470.30	9751.00
Max	5903.89	16,229.13
average revenues per patient in €		
Mean	4189.36	13,847.79
Min	2470.30	3650.36
Max	5903.89	22,434.86
average difference per patient in €		
Mean	433.80	−536.19

## Data Availability

All data are contained within the article.

## Supplementary Materials

The following supporting information can be downloaded at: https://www.mdpi.com/article/10.3390/ebj3010001/s1. Figure S1: Retrospective annual distribution of patients between study and control group. Figure S2: Number of alternative dressing materials and ointment use in the entire control group of patients not receiving Caprolactone (n = 390).
