# Supplementary material for: Paradigm Shift in Treatment Strategies for Second-Degree Burns Using a Caprolactone Dressing (Suprathel®)? A 15-Year Pediatric Burn Center Experience in 2084 Patients
Source: Eur Burn J. 2021 Dec 23;3(1):1–9. doi: 10.3390/ebj3010001 (PMC11575379; doi:10.3390/ebj3010001)
Supplement: Supplementary file 1 [file ebj-03-00001-s001.zip › ebj-1485320-supplementary.pdf]

Supplementary Files:

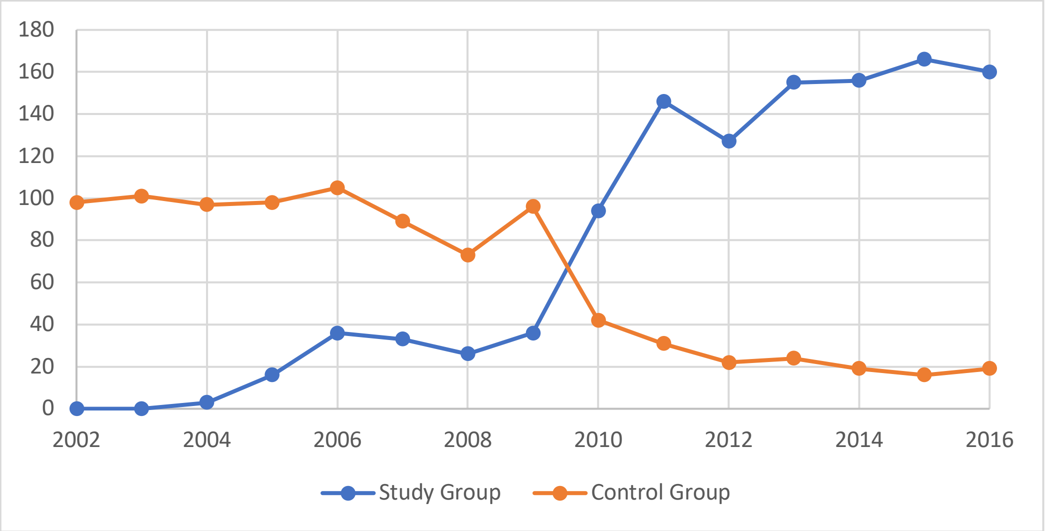

Figure S1: Retrospective annual distribution of patients between study and control group

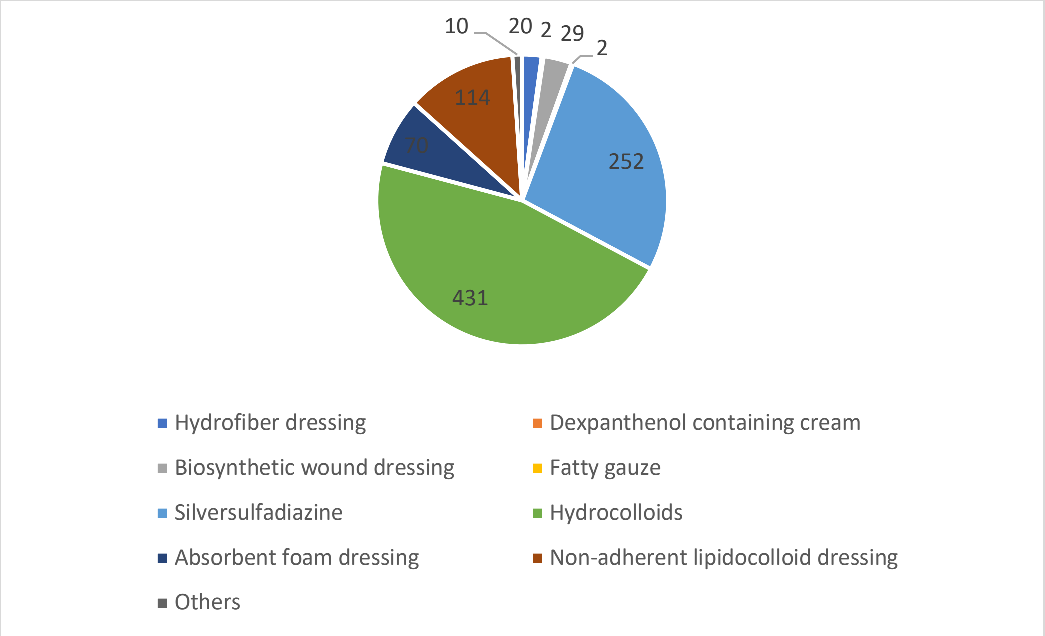

Figure S2: Number of alternative dressing materials and ointment use in the entire control group of patients not receiving Caprolactone (n = 390)
